# Long-term Effects of Regular Whole Blood Donation on Peripheral Blood CD34^+^ Cells Population with using Leukoreduction Filters

**DOI:** 10.30699/IJP.2022.540459.2746

**Published:** 2022-08-16

**Authors:** Parvaneh Abbasi Sourki, Ali Akbar Pourfathollah, Mahdi Pakjoo, Zahra Abbasi-Malati, Mona A. Tajrishi

**Affiliations:** 1 *Department of Hematology, Faculty of Medical Sciences, Tarbiat Modares University, Tehran, Iran*; 2 *Department of Immunology, Faculty of Medical Sciences, Tarbiat Modares University, Tehran, Iran*; 3 *Blood Transfusion Research Center, High Institute for Research and Education in Transfusion Medicine, Tehran, Iran*

**Keywords:** leukoreduction filter, CD34+ hematopoietic stem cells, Regular blood donation, Donor safety

## Abstract

**Background & Objective:**

Trapped cell population in leukoreduction filters (LRFs) contains such a significant number of CD34^+^ hematopoietic stem cells that can be recovered to be used in research studies.

**Methods:**

Samples (n=20) were obtained from 10 first-time donors and 10 regular blood donors with more than 30 times blood donation. After separating leukocytes from LRFs by backflushing, total leukocyte number and differential count were determined in both groups using an automated haemocytometer. Then cell viability and CD34^+ ^cell quantification were assessed using 7- amino-actinomycin D and fluorescent-labeled monoclonal antibodies using flow cytometry, respectively.

**Results:**

Total leukocyte count was 665±164.92×10^6^ in the first-time blood donors and 883±233.89×10^6^ in the regular donors, which were not significantly different (*P*=0.08). While the number of CD34^+^ cells was significantly reduced in the regular donors compared to the first-time donors (0.58±0.20×10^6^/µL vs*. *0.36±0.22×10^6^/µL; *P*=0.034). There was no significant difference in terms of absolute neutrophil count (10.58±3.66×0^6 ^vs. 13.17±6.45×10^6^/µL; *P*=0.349), lymphocytes (7.75±3.11×10^6 ^vs. 10.38±3.77×10^6 ^/µL; *P*=0.917), and monocytes (2.31±0.88×10^6 ^vs. 2.59±1.09×10^6^/µL; *P*=0.591) between the first-time and regular donor groups, respectively. Based on the correlation coefficients, the participants’ age had no significant effect on these variables.

**Conclusion:**

The results of this study depicted that regular blood donation reduces the number of CD34^+^ cells in the peripheral blood (PB) of regular donors while it has no significant effect on the ratio of myeloid to lymphoid cells of the two groups.

## Introduction

Blood products are indispensable part of therapy in many emergencies like accidents, natural disasters, and some genetic disorders (e.g., β-thalassemia major) (1, 2). Voluntary non-remunerated donation of blood from low-risk donors is mostly recommended to supply the needed blood products of healthcare centers (3-5). Leukoreduction filters (LRFs, also known as leukodepletion filters) have been shown to significantly decrease the immunomodulatory effects of transfusion, as well as reducing the possibility of transmission of leukocyte-related infections, particularly, Epstein-Barr virus (EBV), cytomegalovirus (CMV), and human T-lymphotropic virus-I/II (HTLV)-I/II (2, 6-9). Leukodepletion filters, in addition to the functional white blood cells (WBCs), are proved to contain other active cells like dendritic cells (DCs), endothelial progenitor cells (EPCs), hematopoietic stem cells (HSCs), platelets, and red blood cells (RBCs); and as a buffy coat, they have been shown to effectively enhance the in-vitro platelet production (6, 10-15). 

It has been shown that the average number of cluster of differentiation 34 positive (CD34^+^) cells in LRFs reaches 0.4-1.6×10^6^ in each 400-mL blood bag which offers a great opportunity in research around donor hemovigilance while they are usually discarded (7, 16, 17). HSCs and hematopoietic progenitor cells (HPCs) replenish all specialized blood cells during whole life and are recognized and isolated using CD34 glycoprotein marker from bone marrow (BM), umbilical cord blood (UCB), and peripheral blood (PB) by different methods (7, 18-22). LRFs are shown to be highly potential in the stem cell research area, nonetheless, their application in hemovigilance has not yet been investigated. 

Regular blood donation not only provides blood units for patients with the need for blood products like thalassemia, but also it has been shown that donated units by regular donors are safer compared to the units donated by first-time blood donors (3, 23, 24). However, the impact of long-term donation on the quantity and quality of circulating peripheral blood stem cells has not yet been investigated.

Considering that the effects of regular blood donation on the population and viability of HSCs of donors are not known, it is important to investigate whether regular blood donation has any adverse effect on the quantity and/or quality of HSCs and blood cells. Here, we took the advantage of LRFs to determine the effects of long-time regular blood donation on the CD34^+^ cell population and viability of the PB of first-time and regular blood donors.

## Material and Methods

**Subjects**

Participants were selected from those who attended Vesal Blood Transfusion Center, Tehran, Iran. We provided the donors with a questionnaire, accompanied by an information form. Ethical approval was obtained from the National Institute for Medical Research Development (NIMAD, approval number: IR-NIMAD-REC.1396.349). In all procedures described in this article, we followed the ethical guidelines provided by Iranian Blood Transfusion Organization. 

**Leukocyte Recovery from LRFs**

The blood bags were stored at room temperature for 2 hours and filtration was done within 8 hours after collection. The cells were isolated from LRFs and analyzed immediately after filtration. A total of 20 used Leukoflex LCR-5 (Maco Pharma Company, Alborz, Iran) was divided into two groups, including LRFs from 10 first-time blood donors and 10 regular donors with more than 30 times blood donations. LRFs were kept at 4ºC before filtrating and then filtration was carried out at room temperature for 30 minutes by gravity flow. Afterward, the cells were isolated from LRFs using backflushing in a total volume of 200-mL elution buffer using a 60mL sterile syringe filled with phosphate buffer saline (PBS; pH: 7.2) containing 5-mM EDTA and 2.5% sucrose. To remove the remained RBCs, the isolated cells were washed twice using slow centrifugation at 300×g for 10 minutes, and then the pellets were resuspended in PBS.

**Quantification of the Viable Recovered Cells**

Cell counting and survival rate were measured by a hemocytometer (Sysmex Xs-800i, Kobe, Japan) and 7-amino-actinomycin D (7-ADD) staining, respectively.

**Determination of CD34** ^+^ **Cell Population**

To evaluate the immunophenotypic markers by flowcytometry, a cell suspension (10^5^ cells in each sample) was combined with 10 µL of fluorescently-labeled monoclonal antibodies (CD34/PE, and CD45/FITC; DAKO Company, Carpinteria, California) and incubated in dark for 30 minutes at 4ºC. After lysing RBCs by FACS lysing solution, the cells were washed with 500 µL of PBS and fixed with 1% paraformaldehyde. Finally, they were immunophenotyped using Partec PAS III flow cytometer and FlowJo software. 

**Data Analysis**

Data were analyzed using the SPSS software version 24 (SPSS Inc., Chicago, IL, USA) and the results were expressed as means ± standard deviations of triplicates. The normality of the data distribution was evaluated by the Shapiro-Wilk test. Differences between the groups were tested by the student’s t-test after Levene’s test for the equality of variances. The correlation test of age on the numbers of CD34^+ ^HSCs and WBCs were assessed by calculating Spearman's and Pearson's coefficients. Flow cytometry results were analyzed using FlowJo software and immunophenotypic data were analyzed using Partec software. In all analyses, P-values <0.05 were considered statistically significant and data were plotted by the GraphPad Prism software version 7.00 (GraphPad Software, La Jolla California USA) and SPSS software**.**

## Results

**Characteristics of Subjects**

The basic information about donors is summarized in Table 1. Since women with more than 30 times donations were rare, they were excluded from both groups before donation. A total of 20 subjects were then enrolled, including 10 first-time donors (aged 20-57 years old with a median weight of 86.3 kg [range: 55-108 kg] and hemoglobin level of 12-17 g/dL) and 10 donors with more than 30 times donations (aged 41-56 years old with a median weight of 90.8 kg [range: 79-130 kg] and hemoglobin level of 14.5-16 g/dL). 

**Table 1:** The basic information of participants

Regular donors (>30 times)	First-time donors	
**90.8 (79-130)**	86.3 (55-108)	**Weight (kg)**
**49.5 (41-56)**	33.8 (20-57)	**Age (years)**
**462 (453-471)**	465 (450-476)	**Blood volume (mL)**
**15.35 (14.5-16)**	14.23 (12-17)	**Hemoglobin (g/dL)**

**Effect of Blood Donation Frequency on Derived Leukocytes from LRFs**

By comparing the number of derived cells from LRFs of first-time and regular blood donors, a significant difference was observed between the two groups regarding the number of CD34^+^ cells (Table 2). The number of CD34^+^ cells in the regular blood donors was 0.36±0.22×10^6^/µL (*P*=0.034), which significantly reduced compared to their numbers in the first-time blood donors (0.58±0.20×10^6^/µL, *P*=0.034). The number of CD34^+^ cells was not normally distributed among the regular blood donors (*P*=0.001; Table 3). Moreover, the average number of recovered WBCs from LRFs in the first-time and regular blood donors were 665±164.92×10^6^ and 883±233.89×10^6/^µL, respectively, which were not significantly different (*P*=0.08). In addition, there were no significant difference between the first-time and regular donors in terms of the number of neutrophils (0.58±3.66×0^6 ^vs. 13.17±6.45×10^6^/µL; *P*=0.349), lymphocytes (7.75±3.11×10^6 ^vs. 10.38±3.77×10^6 ^/µL; *P*= 0.917), and monocytes (2.31±0.88×10^6 ^vs. 2.59±1.09×10^6^/µL; *P*= 0.591), respectively. 

The viability of isolated leukocytes was determined for both groups after backflushing using the 7-ADD staining. The viability percentage of isolated leukocytes from the first-time blood donors was 97.2±1.13 in comparison with 94.6±2.71 from the regular donors, which did not have a normal distribution based on Shapiro-Wilk test (Table 3). No significant difference was observed between the two groups (*P*=0.02) in terms of viability of isolated leukocytes.

**Table 2:** Equalities of variances and means of each parameter between the two groups were tested by Levene’s test and Independent Sample Test, respectively

**Independent Sample Test**										
**Variables**		Levene's Test for Equality of Variances		t-test for Equality of Means						
		F	P-value	t	df	Sig. (2-tailed)	Mean difference	Std. Error Difference	95% confidence interval of the Difference	
									Lower	Upper
**Recovered WBC count**	Equal variances assumed	1.236	**0.281**	-1.85	18	0.08	-168.1	90.502	-358.238	22.038
	Equal variances not assumed	-	-	-1.85	16.175	0.082	-168.1	90.502	-359.787	23.587
**LRFs CD34** ^+^ ** cells**	Equal variances assumed	0.089	**0.768**	2.28	18	**0.034**	0.22	0.0960	0.0180	0.421
	Equal variances not assumed	-	-	2.28	17.758	0.035	0.22	0.0960	0.017	0.422
**Recovered Neutrophils per µl**	Equal variances assumed	2.368	**0.148**	-0.97	13	0.349	-2.588	2.662	-8.339	3.163
	Equal variances not Assumed	-	-	0.93	9.223	0.373	-2.588	2.762	-8.815	3.638
**Recovered Lymphocytes per µl**	Equal variances assumed	0.0130	**0.910**	0.10	13	0.917	0.204	1.921	-3.945	4.355
	Equal variances not assumed	-	-	0.10	12.618	0.917	0.204	1.925	-3.967	4.377
**Recovered Monocytes per µl**	Equal variances assumed	0.349	**0.565**	0.55	13	0.591	0.281	0.511	-0.822	1.386
	Equal variances not assumed	-	-	0.54	11.572	0.597	0.281	0.518	-0.853	1.417

*The significant variables are in bold from

**Table 3:** Normality of the data distribution using Shapiro-Wilk test

	First-time donors			**R**egular donors (>30 times)		
Parameters	Shapiro-Wilk			Shapiro-Wilk		
	Statistic	Sig.	Normality	Statistic	Sig.	Normality
WBC	0.908	0.269	Normal	0.955	0.724	Normal
CD34^+^cells	0.920	0.357	Normal	0.682	0.001	Non-normal
Neutrophils	0.913	0.374	Normal	0.870	0.151	Normal
Lymphocytes	0.934	0.552	Normal	0.909	0.347	Normal
Monocytes	0.928	0.495	Normal	0.949	0.697	Normal
Viability	0.825	0.029	Non-normal	0.818	0.024	Non-normal
Age	0.883	0.143	Normal	0.945	0.613	Normal

**The Characteristics of Isolated Leukocytes from LRFs**

The isolated leukocytes were evaluated using the surface markers of CD34 and CD45. As shown in R1 regions of figures 2A and 2B, the expression percentages of CD45^+^ cells were 0.081% and 0.055% in the first-time and regular blood donors, respectively. The proportion of CD34^+^ cells was 21.68% and 5.75% of recovered CD45^+^ cells in the first and second groups, respectively (as shown in R2 regions of Figures 2A and 2B). The proportion of CD34^+^/CD45^dim^ cells are shown in R3 regions which were 17.32% and 17.11% of recovered cells in the two groups, respectively. 

**Correlation coefficients between the Age of Subjects and Other Variables**

Spearman's and Pearson's coefficients were used to investigate the correlation between the age of the subjects and other variables in accordance with the types of distribution (as represented in Table 2). There was no significant relationship between the age and other variables (Table 4). 

**Table 4:** The correlation coefficients between the age of the subjects and other variables

	First-time donors		Regular donors (>30 times)	
Parameters	Correlation coefficients		Correlation coefficients	
	Statistic	Sig.	Statistic	Sig.
WBC	-0.491	0.149	0.310	0.383
CD34^+^cells	0.129	0.731	-0.281	0.431
Neutrophils	-0.275	0.509	0.531	0.220
Lymphocytes	-0.447	0.267	-0.089	0.849
Monocytes	-0.236	0.574	0.590	0.163
Viability	-0.129	0.722	0.254	0.479

**Fig. 1:**
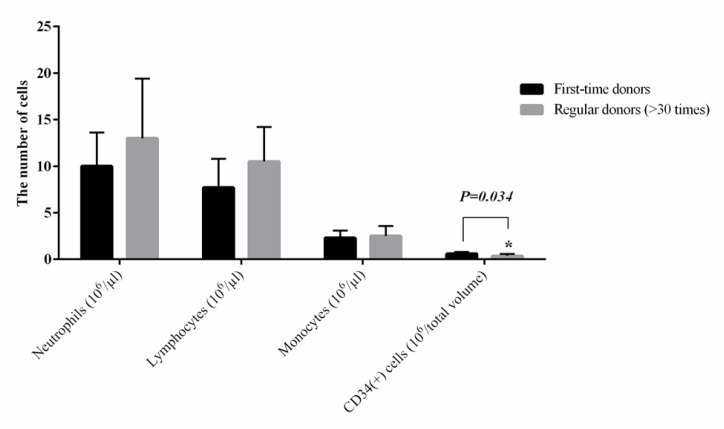
The number of recovered leukocytes isolated from LRFs of the first-time and regular donors according to the type of cells. The results (means ±standard deviations) only comparatively represent a significant difference between the two groups in terms of the number of CD34^+^ cells (^*^P˂ 0.05)

## Discussion

Previous studies have majorly evaluated the effects of regular blood donation on hematopoiesis through the measurement of iron-associated blood parameters and have introduced iron deficiency as its main side effect (18, 25). Although one study has reported that there is no significant difference between the number of CD34^+^ cells isolated from donors before and immediately after a blood donation, it was a short-term study and could not predict long-term effects of blood donation (26). Here, we analyzed the data related to donors whose health was determined by regular examinations in the transfusion centers before donation and showed that there was 1.19-fold reduction in the number of CD34^+^ cells from the regular donors while the myeloid to leucocyte (M/L) ratio did not significantly change (Table 3). In addition, the decreased number of leukocytes had no adverse effect on donors as they were diagnosed healthy and ready to donate by physicians of transfusion centers according to the universal guidelines.

The number/proportion of PB-CD34^+^ cells, myeloid cells, and lymphocytes is imbalanced in many of disorders like leukemia and bone marrow (BM) 

fibrosis versus the healthy condition in which the number of PB-CD34+ cells decrease, and the number of myeloid and lymphoid cells and BM-HSCs remains constant as people age (9, 27-29). Hence, measurement of the quantity and viability of CD34+ cells, total leukocyte number, their differential number, and M/L ratio allow us to monitor significant changes in the number or ratio of WBCs to predict possible hematopoiesis failure following regular donation. However, the differences among mentioned parameters between the first-time and regular donors were not significant and the percentage of viable CD34^+^ cells was more than 90% in both groups. 

It was initially hypothesized that the reduction in LRF-CD34+ cells was correlated with the age, however the statistical analyses revealed that there was no relationship between the measured parameters in this study and the age of participants so that the loss of sensitivity to PB stimuli or any reduction in mobilizing cytokines might be the possible mechanisms of this decrease (28-32). 

**Fig. 2:**
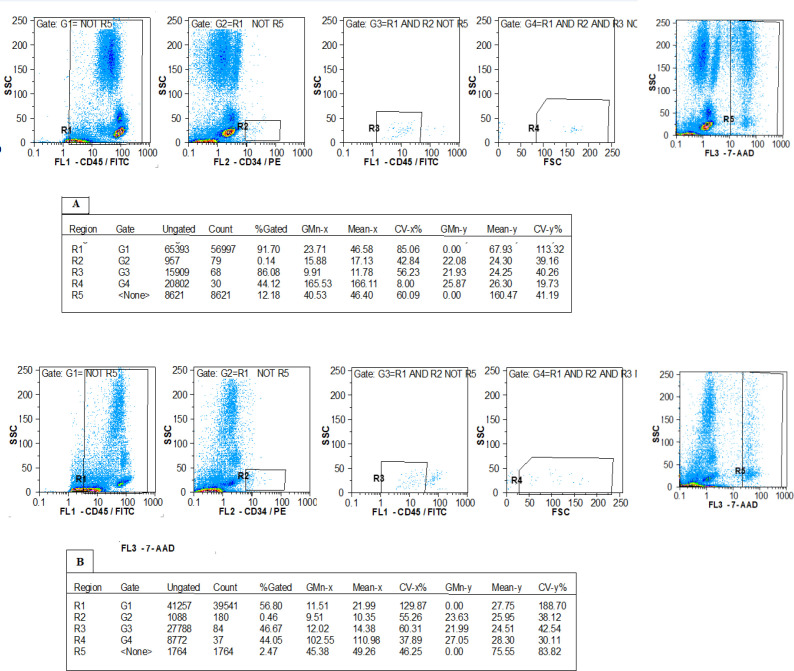
Cell viability and immunophenotypic characterization of CD34^+^/CD45^+^ cells isolated from LRFs of (A) first-time donors and (B) regular donors

## Conclusion

In this study, a decrease in LRF-CD34+ cell count was seen while no adverse effect on the number or function of leukocytes was seen. This study was not a cause-and-effect study and donor clinical parameters were not the target of the study, however, all the donors were diagnosed healthy with routine principals of blood donation at transfusion centers.

## Conflict of Interest

The authors declare no conflict of interest.
